# Influence of Emerging Semiconductive Nanoparticles on AC Dielectric Strength of Synthetic Ester Midel-7131 Insulating Oil

**DOI:** 10.3390/ma15134689

**Published:** 2022-07-04

**Authors:** Muhammad Fasehullah, Feipeng Wang, Sidra Jamil, Muhammad Shoaib Bhutta

**Affiliations:** 1 State Key Laboratory of Power Transmission Equipment & System Security and New Technology, Chongqing University, Chongqing 400044, China; dr.mfk@cqu.edu.cn; 2 Chongqing Key Lab for Advanced Materials and Clean Energies of Technologies, School of Materials and Energy, Southwest University, Chongqing 400715, China; sidrajamil@swu.edu.cn; 3 School of Electronics and Information Engineering, Wuxi University, Wuxi 214105, China; shoaibbhutta@hotmail.com

**Keywords:** AC dielectric strength, nanofluids, solid/liquid interface, bandgap, specific surface area, electrical double layer, thermal analysis, statistical analysis

## Abstract

Exploring impressively effective dielectric nanofluids for transformers to improve dielectric strength and thermal stability is indispensable. It is crucial to determine the modification mechanism of dispersed nanomaterials in insulating oil for operative applications in power transformers. This paper aspires to authenticate the experimental evidence of the enhancing AC dielectric strength of synthetic ester Midel-7131 using two newly introduced semiconductive nanoparticles, CdS and Co_3_O_4_, and uncover the potential reasons for enhanced AC dielectric strength. The AC breakdown voltage (BDV) of synthetic ester and nanofluids was investigated and statistically evaluated. The mean AC breakdown voltage of SE/CdS and SE/Co_3_O_4_ was increased by 31.9% and 31.3%, respectively. The augmentation in AC breakdown strength is possibly due to the facilitated charge-scavenging ability owing to the large specific surface area and wide bandgap. Simultaneous thermogravimetric analysis, differential scanning calorimetry, and derivative thermogravimetry analyses (TGA–DSC–DTG) confirmed that the initial decomposition temperature was high and heat dissipation was low, indicating that the nanofluids were thermally stable in both air and nitrogen. Hence, emerging semiconductive CdS and Co_3_O_4_-based nanofluids of synthetic ester possess remarkable dielectric strength and thermal stability enhancement for their application in power transformers.

## 1. Introduction

The power transformer is the most critical equipment in power systems, playing an essential role in power transmission, distribution, and utilization [1]. It has been reported in the literature that around 75% of transformers collapse due to failure in the insulation system [2,3,4]. Thus, failure of the transformer primarily occurs due to the insulating system’s disintegration [5]. Mineral oil is widely used as an insulating oil because of its good dielectric strength, low viscosity, cooling ability, and high thermal stability. However, considering environmental issues such as safety and limited fossil fuel resources, ester-based oils have attracted tremendous research attention to replace mineral oil because of their advantages, such as higher flame resistance, higher flash and fire points, better biodegradability, and better moisture tolerance [1,6,7]. Furthermore, ester-based oils are beneficial for transformers because they have been proven to increase the lifetime and loading capability [8,9]. Unfortunately, existing dielectric liquids, e.g., mineral oils and ester-based oils, suffer some limitations regarding electrical and thermal performance during operation in power transformers. Hence, the electrical, thermal, and economic concerns should be addressed for improved operation of power transformers [10,11].

The term “nanofluid” was familiarized by Choi et al. in 1995. He dispersed metal oxide nanoparticles into bare fluid and reported enhanced thermal conductivity owing to an improved heat transfer coefficient [12]. However, the improved physiochemical properties of nanotechnology-based insulating nanofluids have provided a new direction to improve the dielectric strength and thermal stability of insulating oils [7,13,14,15]. The most frequently used nanoparticles in insulating oils, such as SiO_2_ [16,17], Al_2_O_3_ [18,19,20], TiO_2_ [1,21], ZnO [22,23], Fe_3_O_4_ [16,24], SiC [18], C_60_ [25,26,27], BN [28], AlN [29], and graphene [30,31], can improve the dielectric strength and thermal stability of insulating oils. To prepare the finest nanofluids, it is also necessary to consider the thermal stability of nanofluids to fulfill the obligation of heat transfer in transformers and to enable ester-based oils to retain their viscosity at higher temperatures [32]. Notably, a minor enhancement of the thermal properties of nanofluids is beneficial for a cost-effective and efficient heat transfer process. It has been reported that the stable dispersion of nanoparticles with high thermal conductivity can significantly enhance the thermal stability of insulating oil-based nanofluids [33].

The electrical breakdown mechanism is immensely complex. It primarily depends upon the nature of bare fluid, i.e., relative permittivity, temperature, water content, and viscosity, while the respective nanoparticle size, specific surface area, bandgap, electrical conductivity, permittivity, and morphology also have an impact [34]. In addition to the aforementioned factors, sample preparation methods, measurement techniques following standard protocols, and purity are influential. The bare fluid–nanoparticle interfacial region is highly influential in enhancing the electrical breakdown by acting as a charge scavenger. Thus, the surface properties primarily influence the occurrence of electrical breakdown in insulating oil-based nanofluids [35,36,37,38]. Usama et al. illustrated that synthetic ester-based nanofluids based on Fe_3_O_4_, Al_2_O_3_, and SiO_2_ could enhance the AC breakdown voltage by 48%, 35%, and 32%, respectively. According to Usama and coworkers, conductive nanomaterials are capable of trapping fast-moving charge carriers and transforming them into slow-moving charge carriers, thereby increasing the breakdown strength [16]. Li and coworkers reported that the AC breakdown voltage of a vegetable oil-based nanofluid based on Fe_3_O_4_ was enhanced by 20% compared to pure oil. The streamer development was evaluated as a function of the charge relaxation time constant “τ”, and it was concluded that Fe_3_O_4_ nanoparticles could affect the electrodynamics of streamer development [24]. Huang and coworkers prepared a vegetable and mineral oil-based nanofluid based on fullerene (C_60_) nanoparticles. It was found that the AC breakdown voltage of the vegetable oil-based nanofluid led to an enhancement of 8.6% at 100 mg/L concentration, while the mineral oil-based nanofluid acquired the optimum breakdown voltage at 200 mg/L, with an enhancement of 21.7% compared to the breakdown voltage of the base oil. Lastly, it was concluded that, considering the electron affinity of C_60_, the molecules of C_60_ captured the free electrons, generating negative ions; as a result, the streamer development was weakened, and the breakdown strength increased [25]. Thus, nanomaterials can significantly enhance the dielectric strength of insulating oils, considering various mechanisms.

In this study, semiconductive nanoparticles, CdS and Co_3_O_4_, were successfully synthesized and then dispersed in synthetic ester (Midel-7131) insulating oil. The phase, morphology, specific surface area, and bandgap were investigated for the as-synthesized nanoparticles. CdS is a II–VI semiconductive compound that possesses a wide bandgap, enabling it to enhance the dielectric strength of transformer oil. In contrast, Co_3_O_4_ is a ferromagnetic material with a main bandgap ranging between 2.1 and 3.2 eV and a sub-bandgap ranging from 1.5 to 1.8 eV. Consequently, Co_3_O_4_ is a semiconductor. These two nanoparticles are emerging semiconductive nano-additives for insulating oil-based nanofluids [39,40]. The AC dielectric strength of CdS and Co_3_O_4_ nanofluids was investigated at four different doping concentrations. A detailed statistical analysis was conducted to inspect the reliability of nanofluids and safe operation of power transformers. The large specific surface area and wide bandgap of these nanoparticles resulted in a nanofluid with improved charge-scavenging ability, thereby enhancing the AC breakdown strength of the bare fluid. Furthermore, the thermal stability of the bare fluid and nanofluids was examined at elevated temperature in air and nitrogen.

## 2. Materials and Methods

### 2.1. Materials

Cadmium chloride hemi-pentahydrate (CdCl_2_·2.5H_2_O), dimethyl sulfoxide (C_2_H_6_OS), cobalt nitrate (Co(NO_3_)_2_), sodium hydroxide (NaOH), and polyoxyethylene sorbitan trioleate (Tween-85) were purchased for the synthesis of nanoparticles.

### 2.2. Synthesis of CdS Nanoparticles

CdS nanoparticles were synthesized using a previously described procedure with a few modifications [39]. Briefly, 0.22 g of CdCl_2_·2.5H_2_O was added to 40 mL of dimethyl sulfoxide under continuous stirring for 60 min. After that, the solution was sealed in a Teflon-lined autoclave and kept at 180 °C for 12 h. Finally, the solution was filtered, washed, and dried at 60 °C for 24 h to obtain the dried CdS nanoparticles.

### 2.3. Synthesis of Co_3_O_4_ Nanoparticles

First, 5 mL of Tween-85 was stirred with 0.3 M sodium hydroxide solution, and then 1 M cobalt nitrate solution was dissolved in the above solution. The solution was continuously stirred at 90 °C for 96 h until it became a black suspension. Finally, 50 mL of ethanol was added. The precipitates were separated using a centrifuge, washed several times, and then dried at 60 °C for 24 h to obtain Co_3_O_4_ nanoparticles.

### 2.4. Material Characterization

In order to observe the morphology, particle size, quantitative elemental composition, and elemental mapping of nanoparticles, high-resolution transmission electron microscopy (HRTEM) was carried out using a Talos F200S (Thermo Fisher Scientific Co., Ltd., Czech Republic). Brunauer–Emmett–Teller (BET) analysis for specific surface area and pore diameter was performed according to N_2_ adsorption using a physical adsorber instrument ASAP-2460 (Mike, Norcross, GA, USA). The phase conformation of the as-synthesized nanoparticles was investigated by X-ray diffraction (XRD) using a Bruker D8. The bandgap of the nanomaterials was calculated according to the absorbance data obtained from UV–visible spectroscopy using a UV-3600 (Shimadzu, Japan). Thermal stability analysis was conducted using a simultaneous TGA–DSC–DTG thermal analyzer 449 F3 (NETZSCH, Germany) to examine the characteristics of thermal stability of the bare fluid and nanofluids at elevated temperatures. This test was performed separately under N_2_ and air atmosphere with a 50 mL/min flow rate. The temperature of the samples was maintained in an isothermal state at 30 °C, and then gradually increased to 600 °C with a heating rate of 10 °C/min.

### 2.5. Preparation of Nanofluids

In this research, a two-step method for the preparation of nanofluids was adopted. Synthetic ester (Midel-7131) insulating oil was filtered using a vacuum filtration setup to remove sludge or impurities with 4.5 µm cellulose filter paper. Two kinds of synthetic ester-based nanofluids were prepared by dispersing the two as-synthesized nanoparticles with four different concentrations of 0.05 g/L, 0.2 g/L, 0.3 g/L, and 0.4 g/L. To deal with possible agglomeration and sedimentation due to magnetic forces, the solution was magnetically stirred for 30 min, followed by 2 h of sonication using a probe-based ultrasonic homogenizer without any surfactant. Furthermore, the sonicated samples were placed into a vacuum oven for degassing and drying for 48 h at 60 °C. Finally, stable as-prepared nanofluids were acquired for testing. Both nanofluids exhibited stability for around 1.5 months, especially at a lower doping concentration.

The physiochemical properties of synthetic ester Midel-7131 insulating oil are tabulated in Table 1.

### 2.6. AC Breakdown Voltage Measurements

AC breakdown voltage measurements of pure synthetic ester and nanofluids were performed by following the IEC 60156 standard using IJJD-80 (Ruixin Electrical Test Equipment Co., Ltd., Wuhan, China). A plate–plate electrode setup was used with an electrode diameter of 25 mm, and the electrode gap was fixed at a 2.5 mm distance. Therefore, AC stress of 50 Hz was applied continuously at a ramp rate of 2 kV/s until breakdown occurred. The parameters used to investigate AC breakdown voltage were as follows: the total number of continuous readings was 8, the magnetic stirring duration was set to 60 s, and the rest period was 90 s to provide time for the oil sample to undergo self-healing. Furthermore, statistical analysis was performed to verify the experimental data following a Weibull distribution and normal distribution in order to test the reliability and safety of the bare fluid and nanofluids.

## 3. Results and Discussions

**Figure 1:**
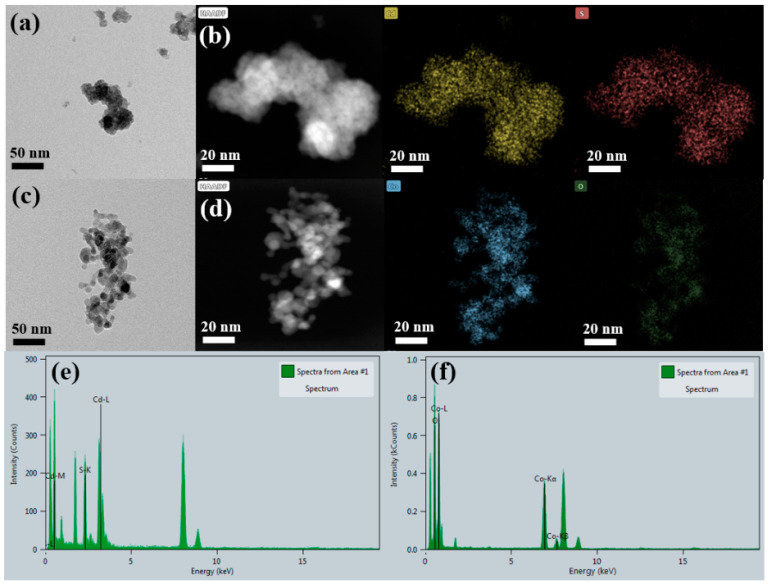
HRTEM images of (**a**) CdS and (**c**) Co_3_O_4_; HAADF images with corresponding elemental mapping of (**b**) CdS and (**d**) Co_3_O_4_; EDS spectrum of (**e**) CdS and (**f**) Co_3_O_4_.

The morphology, particle size, and quantitative elemental composition were confirmed by high-resolution transmission electron microscopy (HRTEM) analysis, as displayed in Figure 1. The HRTEM image of CdS nanoparticles displayed agglomeration; however, the primary grains were spherical with an irregular distribution, as shown in Figure 1a. The high-angle annular dark field (HAADF) image also confirmed the morphology of CdS nanoparticle with corresponding elemental mapping confirming the presence of Cd and S. The average crystallite size was found to be 10 nm. Moreover, the EDS line spectrum illustrated in Figure 1e further confirmed the existence of Cd and S by showing sharp peaks. The HRTEM image in Figure 1c depicts the nano Co_3_O_4_ with dispersed nano-crystallites of spherical morphology with a uniform crystallite size. The average crystallite size was found to be 20 nm. In addition, the EDS elemental mapping of the corresponding HAADF image in Figure 1d also verified the presence of Co and O. Furthermore, the EDS spectrum in Figure 1f illustrates sharp peaks indicating the presence of Co and O. Noticeably, the Co and O atoms exhibited a uniform distribution throughout the sample, indicating the effectual incorporation of Co and O atoms.

**Figure 2:**
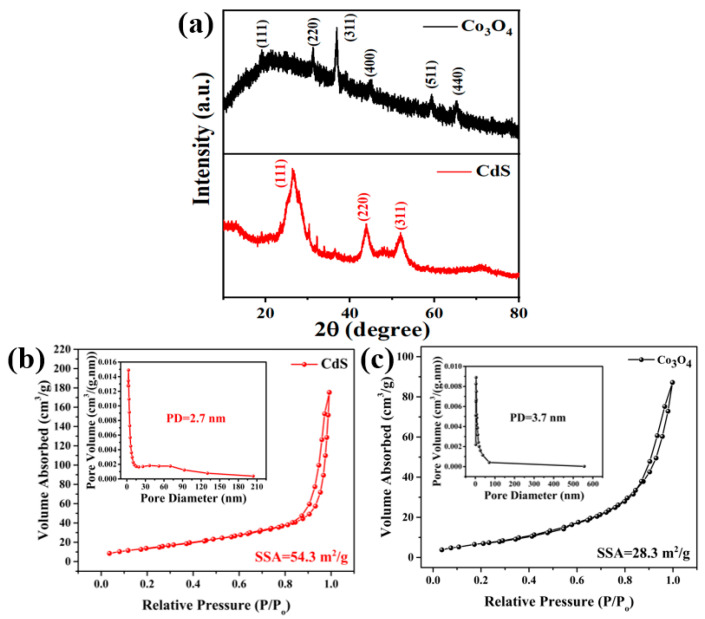
(**a**) XRD spectra of CdS and Co_3_O_4_ nanoparticles; specific surface area with inset displaying pore size distribution of (**b**) CdS and (**c**) Co_3_O_4_.

The phase verification of the as-synthesized samples was performed using X-ray diffraction (XRD) analysis. Figure 2a depicts the XRD pattern of CdS possessing dominant diffraction peaks at 26.45°, 44.1°, and 52.21° corresponding to (111), (220), and (311), ascribed to the zinc blende (cubic) phase of CdS [41]. The remaining peak also matched well with the JCPDS card 75-1546 of cubic CdS. In contrast, the diffraction peaks at 19.11°, 31.23°, and 36.78° were stronger reflections indexed to the (111), (220), and (311) planes of the Co_3_O_4_ crystal structure. All peaks for the Co_3_O_4_ nanoparticles matched well with the cubic spinel structure (JCPDS card 42-1467) [42]. No impurity peak was detected in any of the samples; hence, the synthesized nanoparticles possessed high crystallinity and purity.

A porous nanomaterial with a large specific surface area is advantageous for enhancing the dielectric performance of insulating oil [39,43]. The specific surface area (SSA), pore diameter (PD), and porosity of CdS and Co_3_O_4_ nanoparticles were determined using Brunauer–Emmett–Teller (BET) measurements. The N_2_ adsorption–desorption curves in Figure 2b depict CdS NPs with an SSA of 54.3 m^2^/g with a homogenous pore distribution and an average pore diameter of 2.7 nm. Moreover, Co_3_O_4_ nanoparticles exhibited a specific surface area of 28.3 m^2^/g with a pore diameter of 3.7 nm, as shown in Figure 2c. The high porosity facilitated the generation of a charged surface to interact with insulating oil. All nanoparticles were mesoporous, possessing a well-defined and well-ordered homogenous network with nanochannels. The high specific surface area of nanoparticles triggered the charge-trapping mechanism, thereby improving the AC breakdown strength [39,43,44].

**Figure 3:**
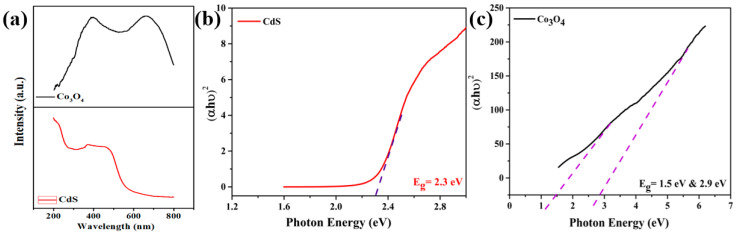
(**a**) UV–Vis spectra of CdS and Co_3_O_4_ and the corresponding Tauc plots of (**b**) CdS and (**c**) Co_3_O_4_.

UV–visible spectroscopy was performed to investigate the bandgap of nanostructures. Figure 3a demonstrates a blueshift with an absorption peak positioned at 484 nm for CdS NPs compared to bulk CdS (525 nm). The blueshift was attributed to the quantum confinement of charge carriers ascribed to the smaller size of CdS NPs [45,46]. However, Co_3_O_4_ displayed dual bands attributed to the O^2−^–Co^2+^ and O^2−^–Co^3+^ states of Co. The bandgap of the nanoparticles was calculated using the following Tauc relationship [39,47]:

(1) $${\left(\alpha h\nu \right)}^{n}=B(h\nu -E_{g}),$$

(2) $$\alpha =\frac{2.303A}{d},$$

where *hv* is the energy of the photon, *B* is a constant, *n* = 2/3 for a directly forbidden gap, 2 for a direct bandgap, and 1/2 for an indirect bandgap, and *α* is the absorption coefficient obtained from Beer–Lambert’s relation (Equation (2)), where *A* is the absorbance and *d* is the path length of the quartz cuvette.

The bandgap of CdS NPs was 2.3 eV, while the bandgaps of Co_3_O_4_ NPs were 1.5 and 2.9 eV (compared to 1.48 and 2.19 eV for bulk Co_3_O_4_) [35].

### 3.1. AC Breakdown Voltage

**Figure 4:**
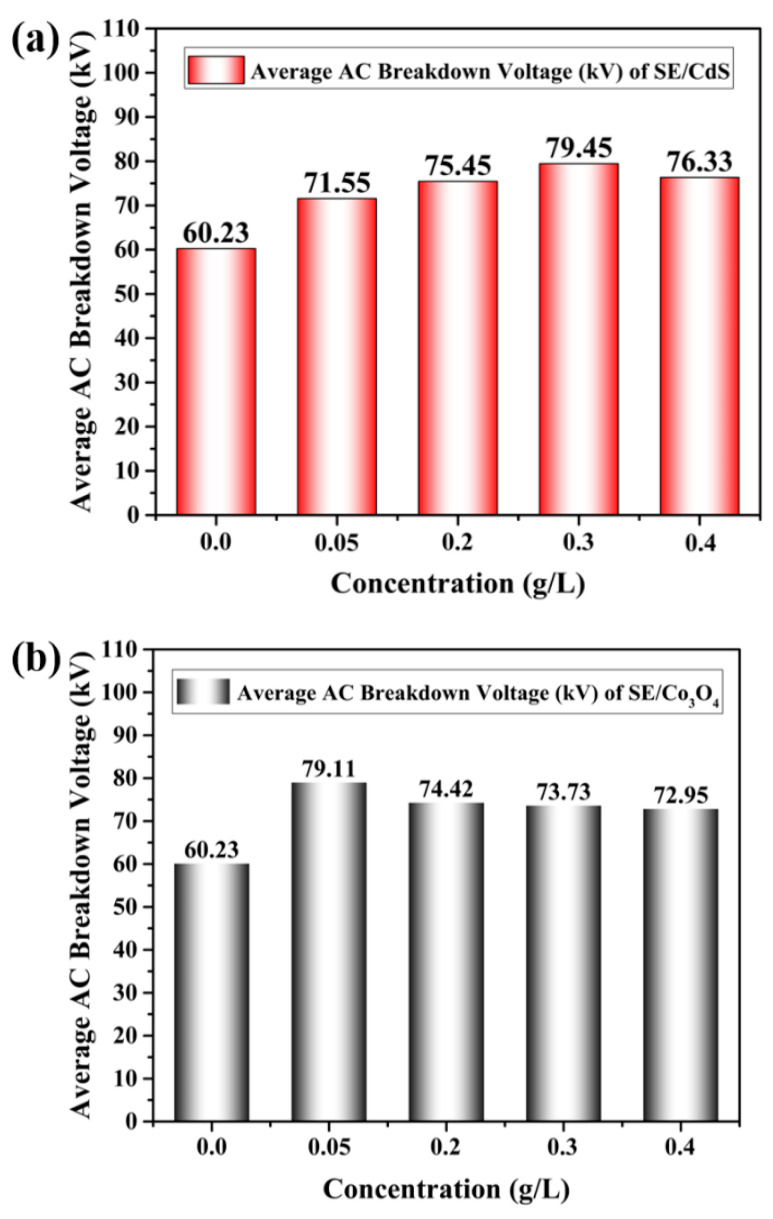

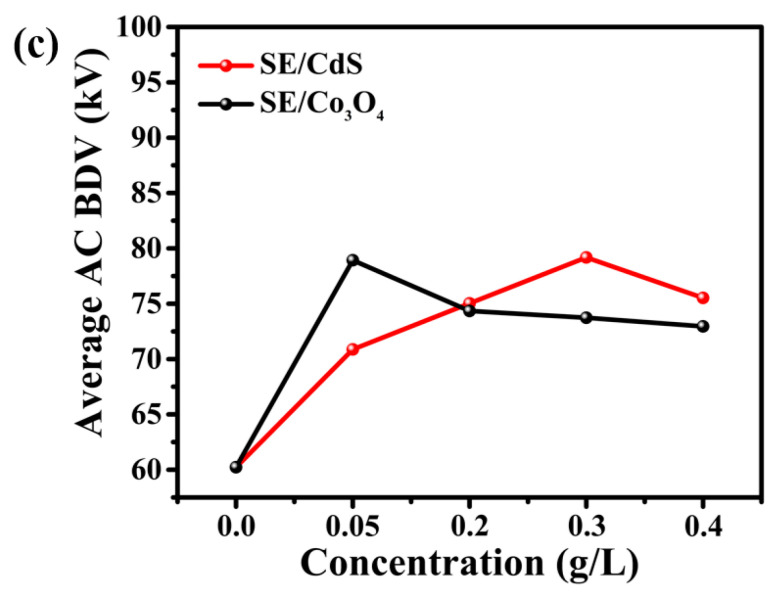
Average AC breakdown voltage (kV) of (**a**) SE/CdS and (**b**) SE/Co_3_O_4_ at different doping concentrations; (**c**) comparison of average AC breakdown voltage of nanofluids.

The mean AC BDV measurements of all nanofluids were compared with the mean AC breakdown voltage of synthetic ester oil, as shown in Figure 4. The results revealed that the AC breakdown voltage of nanofluids significantly increased as compared to pure synthetic ester oil. The AC breakdown voltage was not negatively influenced by the addition of nano-additives at all doping concentrations. According to the results shown in Figure 4a, SE/CdS exhibited an increment in the AC breakdown voltage upon increasing the doping concentration to a certain extent, and then before subsequently decreasing. SE/CdS displayed the highest AC breakdown value of 79.45 kV at an optimum doping concentration of 0.3 g/L, showing an improvement of 31.9% compared to pure synthetic ester oil.

Similarly, SE/Co_3_O_4_ also exhibited a higher average AC breakdown voltage at all doping concentrations. However, SE/Co_3_O_4_ nanofluids evinced the maximum AC breakdown voltage at a doping concentration of 0.05 g/L, before decreasing with a further increase in the doping concentration but remaining higher than the AC breakdown value of base oil. Nevertheless, the average AC breakdown voltage enhancement was 31.3% compared to synthetic ester oil, as displayed in Figure 4b. Hence, it can be deduced that CdS- and Co_3_O_4_-based nanofluids presented considerably enhanced AC breakdown voltage compared to bare fluid. The introduced semiconductive nanomaterials were compatible with synthetic ester oil and capable of enhancing its dielectric strength.

The increase in doping concentration of the nanoparticles led to an increase in the electrical conductivity of dielectric nanofluids until a certain limit, which enabled the development of an uninterrupted path for electron mobility [25,48]; this conclusive doping concentration is called the percolation threshold [25]. The doping concentration of nanoparticles must be lower than the percolation threshold to reduce the charge mobility; otherwise, conduction might occur because of tunneling between nanoparticles due to the small interparticle distance [49]. This is possibly why the AC breakdown voltage declined after a specific doping concentration.

The BDV value of an insulator describes the lowest withstand voltage at which it behaves like a conductor due to the formation of a conducting channel in an insulating medium under specific electrical stress. Such a specific withstand voltage value depicts the dielectric strength of electrical insulation. An insulator might exhibit variation due to different test protocols [50]. The mean AC breakdown voltage and increment percentage (%) of the synthetic ester and nanofluids are presented in Table 2. Additionally, Table 2 illustrates the corresponding breakdown electric field strength (EFS), which was calculated using the following formula:

(3) $$E=\frac{V}{d}$$

where *E* is the electric field strength, *V* is the applied voltage, and *d* is the distance between the two electrodes (2.5 mm in this study).

When an insulating oil is exposed to a high voltage, charge carriers travel quickly toward the opposite electrode due to the formation of a conducting channel between the two electrodes, thereby generating a streamer which leads to electrical breakdown in the insulating oil medium. The formation of streamers is mainly dependent on the applied high voltage, as well as the electric field strength. However, when nanomaterials are dispersed in the insulating oil, the resultant nanofluids perform differently due to an alteration in the electrodynamics of streamers in the insulating oil medium. The dispersion of nanomaterials in insulating oil enables resultant nanofluids to capture fast-moving charge carriers, which delays the relaxation time and streamer propagation, resulting in the improved dielectric strength of insulating oil-based nanofluids [51,52,53]. The nanoparticles in contact with the insulating oil medium attract charge carriers and generate a strong layer named the Stern layer. The diffuse layer is formed around the Stern layer, which attracts more charge carriers until saturation occurs. These layers generate an electrical double layer around the particles [54,55]. The formation of the electrical double layer depends upon various factors, e.g., the high specific surface area of the nanoparticles, high bandgap, difference in electrical conductivity and dielectric constant of the nanoparticles and insulating oil, and surface energy states (i.e., proximity of oxygen vacancy) [21,31].

**Figure 5:**
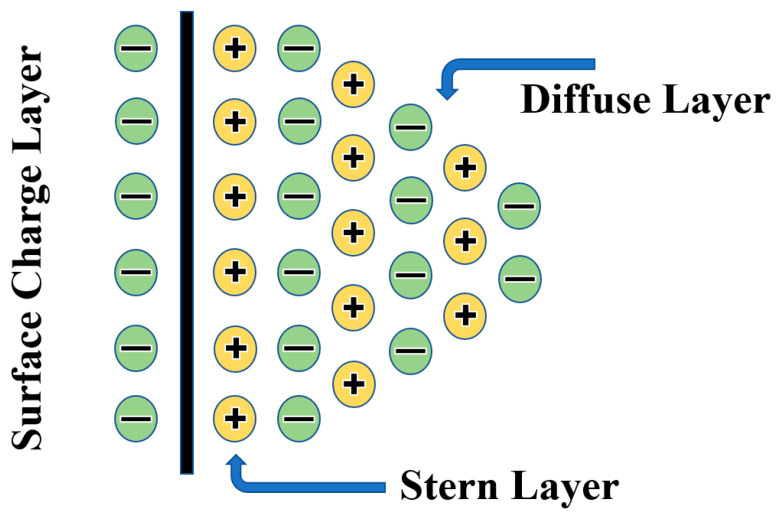
Formation of electrical double layer (EDL) [39].

The dissimilarities in the electrical conductivity and relative permittivity between the insulating oil and nanomaterial generates a discontinuous interfacial region because of the different positions of valence and conduction bands of insulating oil and nanomaterial. For that reason, the electronic band bends toward the interface, which leads to the induction of surface energy states. The aforementioned surface energy states generally consist of various defect states, e.g., interstitial defects or chemical defects, which exist on the surface of nanomaterials. These defect states influence the charge density due to the dispersion of nanomaterials into the insulating oil, resulting in the attraction of charges from the insulating medium to the surface of nanoparticle, which leads to the formation of an electrical double layer (EDL) on the surface of the nanomaterials. In the present work, the improvement in the AC dielectric strength of nanofluids was possibly a result of the charge trapping and de-trapping process due to the formation of an electrical double layer on the surface of the nanoparticles in the insulating medium as shown in Figure 5.

Since the nanoparticle size is inversely proportional to the specific surface area of the nanomaterial, the surface energy and surface binding energy of the nanomaterial will increase upon decreasing the size of the crystallite in the nanomaterial. When nanoparticles with a large specific surface area are dispersed in insulating oil, they generate an electrical double layer at the liquid–solid interface due to the high surface energy of the nanoparticles [21,53,56]. However, it is also evident from the literature that the dispersion of nanoparticles with a wide bandgap in insulating oil enables the resultant nanofluid to capture more electrons due to less repulsive forces from the electron population in the valence band [57], which leads to a delay in streamer propagation, resulting in the augmentation of breakdown voltage, and vice versa. This is evident from the BET and UV–Vis spectroscopy results illustrated in Figure 2 and Figure 3, using nanoparticles with a large specific surface area (CdS = 54.3 m^2^/g; Co_3_O_4_ = 28.3 m^2^/g) and wide bandgap (CdS = 2.3 eV; Co_3_O_4_ = 1.5 and 2.9 eV). Accordingly, the charge density on the surface of the nanomaterials increased with the passage of time until saturation occurred. As a result, nanoparticles were able to repel further incoming charge carriers upon becoming saturated (i.e., all existing sites on the surface of the nanoparticle were occupied). This allowed free charges to be un-trapped, which then moved toward the opposite electrode, causing the breakdown in the insulating oil medium. Hence, in light of the high specific surface area and wide bandgap of the nanomaterials, it is apparent that the AC dielectric strength of the nanofluids was improved due to the formation of a strong electrical double layer on the surface of the nanomaterials.

### 3.2. Statistical Analysis

The most commonly used probabilistic distribution methods to statistically analyze the breakdown voltage of dielectrics are the Weibull distribution and normal distribution. Such tools are advantageous to design and maintain power equipment, as well as enhance the predictability of insulation reliability [58]. The Anderson–Darling [59] and Shapiro–Wilk [60] tests allow verifying the conformity of experimental data with a probabilistic distribution.

The significance level *α* is used to compare the *p*-values in order to check the conformity to a specific probabilistic distribution. It is necessary to recall that, if the *p*-value is less than or equal to the selected alpha value (*α* = 0.05 in this study), then the null hypothesis is considered to be rejected, indicating that the data do not follow a specific probabilistic distribution. A smaller *p*-value is stronger grounds for rejecting the null hypothesis. However, a larger Anderson–Darling value evidences that the data do not follow a Weibull distribution.

#### 3.2.1. Weibull Distribution

The mean AC breakdown voltage allows gauging the dielectric strength of an insulating oil; however, it is insufficient to accurately analyze its efficiency and reliability in transformers. A transformer’s design depends on the lowest withstand voltage of the insulating oil rather than the mean AC breakdown voltage [16]. Therefore, a Weibull probability distribution was used in this work to evaluate the AC breakdown voltage of the pure synthetic ester oil and nanofluids at different breakdown probabilities. The cumulated distribution function of the Weibull probability distribution is given in Equation (4) [1,61,62].

(4) $$F\left(x\right)=1-e^{-\frac{x}{\beta }\alpha },$$

where *α* is a shape parameter, *β* is a scale parameter, and *x* is the breakdown voltage. The shape parameter “*α*” indicates the slope of the line in the probability plot, which influences the shape of the curve, whereas the scale parameter “*β*” is associated with the scattering of data and expresses the degree of failure. However, the Anderson–Darling (AD) value corresponds to the measured area between the fitted line and the empirical distribution function, which depends on the data points. N indicates the total number of breakdowns, while the *p*-value provides evidence against the null hypothesis [16,58].

**Figure 6:**
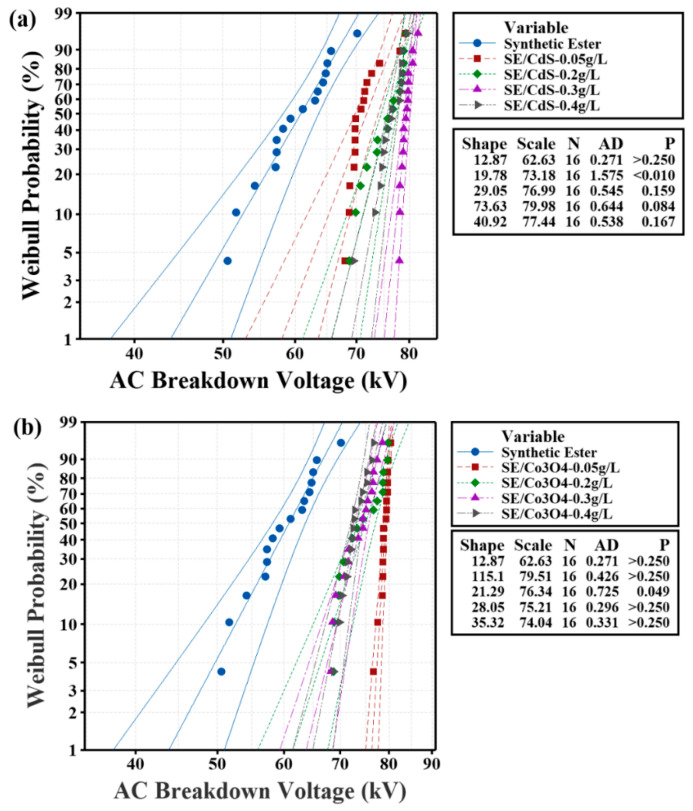
Weibull probability distribution plot of AC breakdown voltages of (**a**) SE/CdS and (**b**) SE/Co_3_O_4_, compared with synthetic ester.

Figure 6a,b exhibit the Weibull probability plots of the synthetic ester and nanofluids. The primary specific parameters such as the shape parameters, scale parameters, Anderson–Darling values, and *p*-values of these curves are indicated in the figure. The *p*-values of the synthetic ester and nanofluids are listed in Table 3, indicating the conformity to a Weibull distribution. It is evident in Table 3 that all samples followed a Weibull distribution except for SE/CdS-0.05 g/L and SE/Co_3_O_4_-0.2 g/L, in accordance with their *p*-values.

The breakdown voltage at low probability (e.g., 1% or 10%) allows determining the minimum possible withstand breakdown voltage of the insulating oil, which is a safety factor when manufacturing electrical equipment [61,63,64]. Therefore, it indicates the reliability of the insulating oil. However, the 50% probability is an indication of the mean breakdown value [16]. The AC BDV and increment percentage at the breakdown probabilities of 1%, 10%, and 50% for nanofluids at all doping concentrations, as obtained from the Weibull probability distribution curves, are shown in the Table 3, compared with the Weibull probability distribution of synthetic ester oil.

At 1%, 10%, and 50% breakdown probability, the AC breakdown voltage of SE/CdS nanofluid at a doping concentration of 0.3 g/L was increased by 71.5%, 47.5%, and 30.8% respectively. However, the SE/Co_3_O_4_ nanofluid at a doping concentration of 0.05 g/L exhibited increments of 74.4%, 48.2%, and 30.2%, respectively, as shown in Table 4.

An exceptional development can be noted in Table 4, whereby the AC breakdown voltage for 1% and 10% probability was considerably higher for optimum doping concentrations as compared to other doping concentrations. This table depicts the 1% and 10% probability for SE/CdS (0.3 g/L) and SE/Co_3_O_4_ (0.05 g/L) as U1 = 75.13 kV and U10 = 77.57 kV with a standard deviation of 1.02 kV, and U1 = 76.39 kV and U10 = 77.97 kV with a standard deviation of 0.92 kV, respectively. The breakdown probability was higher due to the extremely low standard deviation of SE/CdS (0.3 g/L) and SE/Co_3_O_4_ (0.05 g/L) nanofluids, and their performance stability was retained during testing. A higher breakdown voltage value at 1% probability indicates that the insulating oil possesses a stronger ability to withstand electrical stress; for instance, there was only a 1% possibility of breakdown occurring when the high voltage applied on SE/CdS (0.3 g/L) and SE/Co_3_O_4_ (0.05 g/L) nanofluids reached 75.13 kV and 76.39 kV, respectively.

#### 3.2.2. Normal Distribution

**Figure 7:**
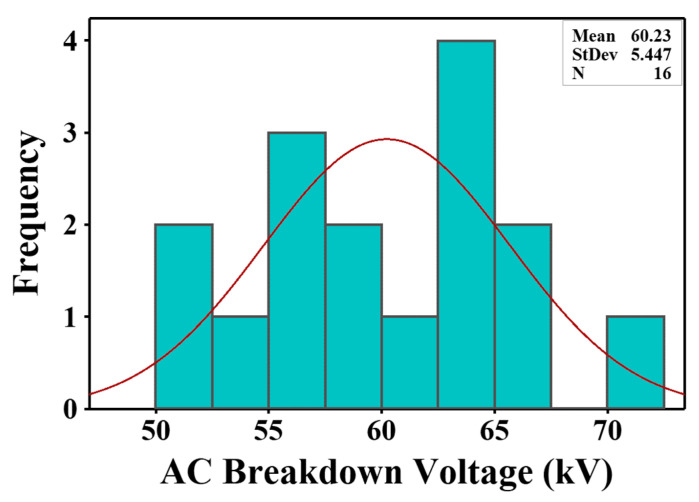
AC breakdown voltage histograms of synthetic ester.

**Figure 8:**
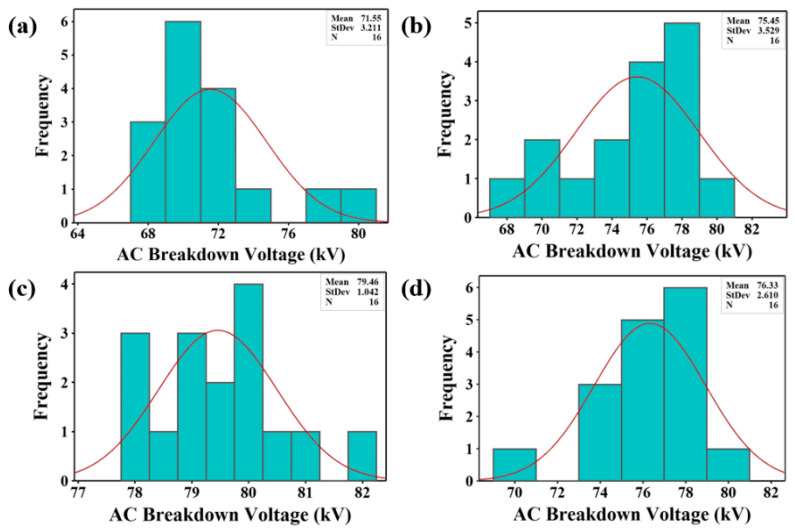
AC breakdown voltage histograms of SE/CdS nanofluids: (**a**) SE/CdS (0.05 g/L), (**b**) SE/CdS (0.2 g/L), (**c**) SE/CdS (0.3 g/L), and (**d**) SE/CdS (0.4 g/L).

**Figure 9:**
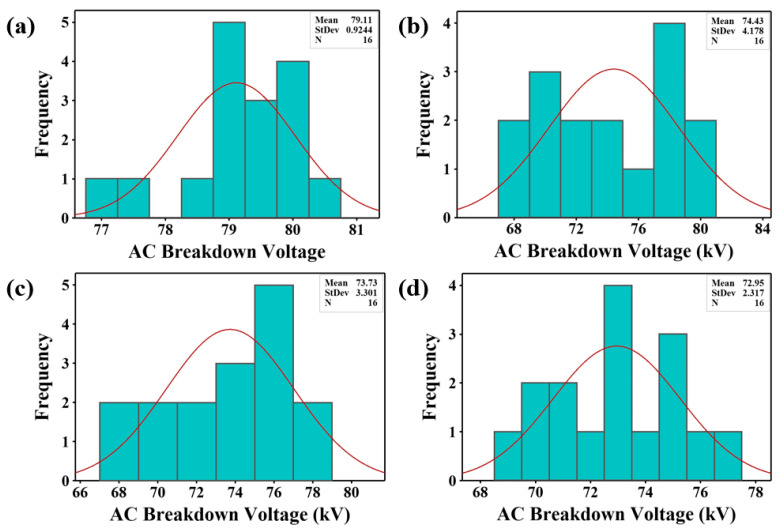
AC breakdown voltage histograms of SE/Co_3_O_4_ nanofluids: (**a**) SE/Co_3_O_4_ (0.05 g/L), (**b**) SE/Co_3_O_4_ (0.2 g/L), (**c**) SE/Co_3_O_4_ (0.3 g/L), and (**d**) SE/Co_3_O_4_ (0.4 g/L).

The histograms of AC breakdown voltage measurements for the bare fluid and nanofluids at various doping concentrations are presented in Figure 7, Figure 8 and Figure 9. The red lines depict the Gaussian curve of the measurements. A narrow range of voltage resulted in a sharper peak of the Gaussian curve due to the smaller standard deviation [65], as shown in Figure 8c,d and Figure 9a,d. On the other hand, an increment in the standard deviation resulted in a flatter and wider Gaussian curve, as shown in Figure 7, Figure 8a,b, and Figure 9b,c. It can be observed that all experimental data followed a normal distribution except for SE/CdS (0.05 g/L), as displayed in Figure 8a.

In order to further approve the conformity, the numerical measurements of shape, i.e., “skewness and kurtosis”, were computed. The skewness specifies the deviation from horizontal symmetry, whereas kurtosis designates the height and sharpness of the Gaussian curve. The skewness and kurtosis values must be within the range of −3 to +3 to verify the normal distribution. Zero values of skewness and kurtosis indicate a standard normal distribution [16].

According to Table 5, all the skewness and kurtosis values were between −3 and +3. The results indicated a deviation from a normal distribution with skewness values ranging from −1.146 to +1.508. Positive skewness values indicate that the distribution is asymmetric to the left of the mean value, away from the fitting curve with a high probability. In contrast, negative skewness values indicate that the distribution is skewed toward the right of the mean value, away from the fitting curve with a low probability. The kurtosis values ranged from −1.719 to +1.683. The highest value of kurtosis was +1.683 for the SE/CdS (0.4 g/L) sample. Positive kurtosis values indicate a leptokurtic distribution whereas negative kurtosis values indicate a platykurtic distribution [66,67].

An Anderson–Darling test was performed using the experimental data in order to test the conformity to a normal distribution by computing the *p*-values. Table 6 presents the *p*-values of synthetic ester and nanofluids, as well as the conformity to a normal distribution. The computed *p*-values of the experimental data were larger than the significance level (α = 0.05) except for the SE/CdS (0.05 g/L) nanofluid sample. Thus, all samples allowed accepting the null hypothesis of conforming to a normal distribution except for the SE/CdS (0.05 g/L) nanofluid sample.

### 3.3. Thermal Stability Analysis

#### TGA–DSC–DTG

**Figure 10:**
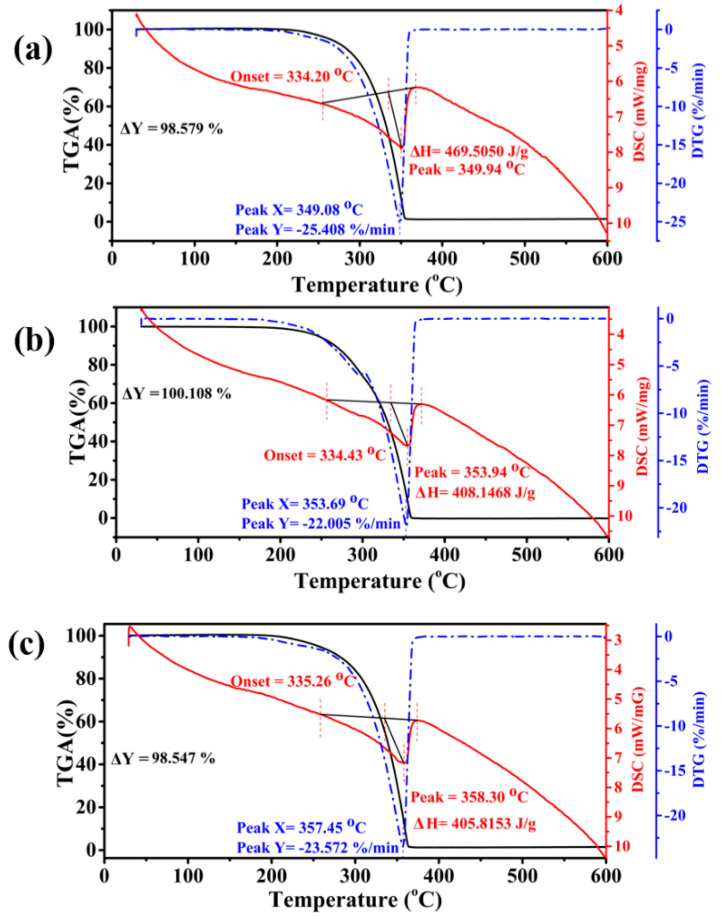
TGA−DSC−DTG curves of (**a**) pure synthetic ester, (**b**) SE/CdS, and (**c**) SE/Co_3_O_4_, at a heating rate of 10 °C/min in N_2_.

Another critical index when analyzing the properties of nanofluids is the thermal stability for use in high-voltage applications [6,68]. Thermal degradation is one of the leading causes of failure of insulating oil when subjected to elevated temperatures [68,69]. Figure 10a depicts the thermal behavior of pure synthetic ester in nitrogen (N_2_). The onset temperature indicates the initial decomposition (334.2 °C for pure synthetic ester). Usually, a lower onset temperature results in the oil degrading and burning out into hydrocarbons at an earlier timepoint [70]. Oxidation starts at the onset temperature; with a further increase in temperature, the mass loss (%) drastically increases due to the volatilization of alkanes and methyl esters [71]. The detailed overview of TGA–DSC–DTG test results is tabulated below in Table 7.

In contrast, Figure 10b,c display the thermogravimetric analysis (TGA) curves with onset temperatures of 334.43 and 335.26, respectively. Herein, we can conclude that the pure synthetic ester and all nanofluids possessed almost similar onset temperatures in N_2_. Furthermore, the differential scanning calorimetry (DSC) analysis revealed the thermo-oxidative degradation of pure oil and nanofluids. The DSC curve for pure synthetic ester oil in Figure 10a displayed an endothermic peak at 349.94 °C with heat absorption of 469.50 J/g. In contrast, the SE/CdS nanofluid with a doping concentration of 0.3 g/L exhibited an endothermic peak at 353.94 °C with heat gain of 408.14 J/g, ascribed to the enhanced oxidative stability after the insertion of CdS nanoparticles. In comparison, the endothermic peak was 358.30 °C for SE/Co_3_O_4_ (0.05 g/L) nanofluid with heat absorption of 405.81 J/g. The endothermic peak denotes the breakage of bonds resulting in the release of gases [72]. Nanofluids exhibited the greatest reduction in thermo-oxidative degradation with the highest endothermic peak temperatures. The results indicate that the dispersion of nanomaterials significantly increased the endothermic temperature of all nanofluids compared to the pure synthetic ester oil, owing to the improved decomposition-resistive characteristics of nanofluid compared to pure oil. The enhancement of thermal properties can be ascribed to the relative contents of saturated long-chain hydrocarbons. Notably, the SE/Co_3_O_4_ nanofluid showed the least volatilization according to the heat gain value (405.8153 J/g), highlighting it as a stable nanofluid with improved thermal stability.

**Figure 11:**
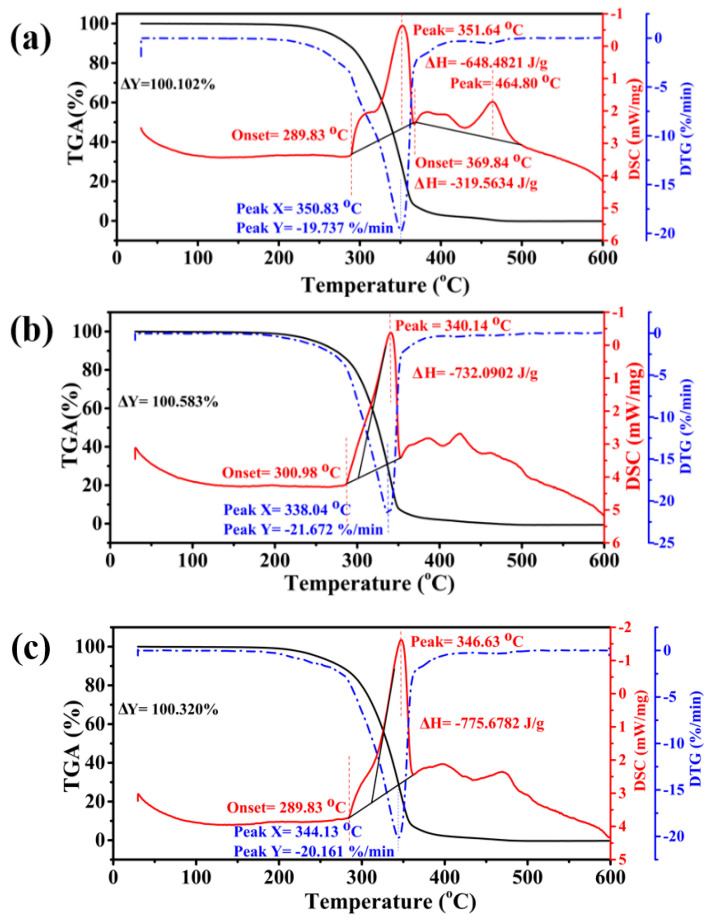
TGA−DSC−DTG curves of (**a**) pure synthetic ester, (**b**) SE/CdS, (**c**) and SE/Co_3_O_4_ at a heating rate of 10 °C/min in air.

Derivative thermogravimetry (DTG) curves can describe the degradation mechanism by illustrating the mass loss with increasing temperature [73]. The DTG curves in Figure 10c display a weight loss rate of 25.408%·min^−1^ for pure synthetic ester oil at 349.08 °C, whereas the SE/CdS nanofluid had a DTG peak at 353.69 °C with a loss rate of 22.005%·min^−1^. Furthermore, the SE/Co_3_O_4_ nanofluid presented a DTG peak at 357.45 °C with a weight loss rate of 23.572%·min^−1^. The DTG results suggest that the oxidation of pure synthetic ester was initiated at a lower temperature compared to the nanofluids. Specifically, SE/CdS and SE/Co_3_O_4_ nanofluids exhibited enhanced thermal stability, attributed to the DTG peaks at relatively higher temperatures than the pure synthetic ester oil. To explore the thermal stability in detail, thermal analysis was also carried out in air. The initial decomposition temperature marked as the onset temperature was 289.83 °C with complete oxidation at 369.84 °C for pure oil. However, the burnout temperature was 300.98 °C for SE/CdS and 289.83 °C for SE/Co_3_O_4_ nanofluid, as displayed in Figure 11. Notably, the onset temperature was the highest for SE/CdS nanofluid in air. The newly introduced SE/CdS nanofluid displayed a stable thermal performance compared to the pure synthetic ester and other nanofluids in both N_2_ and air. Moreover, the DSC curves in Figure 11a reveal its exothermic peak at 351.64 °C; however, with a further increase in temperature, another exothermic peak appeared at 464.80 °C with complete decomposition. In comparison, Figure 11b displays an exothermic peak at 340.14 °C for SE/CdS nanofluids, while the exothermic peak of SE/Co_3_O_4_ nanofluid appeared at 346.63 °C, as shown in Figure 11c. The nanofluids exhibited excellent thermo-oxidative stability with a single exothermic peak compared to the pure oil. These results indicate that the exothermic peaks emerged at slightly higher temperature for pure synthetic ester oil; however, the heat dissipation was greater for pure synthetic ester oil, whereas the nanofluids exhibited reduced heat generation due to the breakage of bonds, signifying their improved thermal stability.

The DTG curves in Figure 11 allowed investigating the degradation mechanism with a weight loss rate of 19.737%·min^−1^ for pure synthetic ester oil at 350.83 °C, whereas the SE/CdS nanofluid had a DTG peak at 338.04 °C with a loss rate of 21.672%·min^−1^. Notably, the SE/Co_3_O_4_ nanofluid presented a DTG peak at 344.13 °C with a loss rate of 20.161%·min^−1^. The DTG results indicate the degradation of pure synthetic ester oil at a slightly higher temperature than that for nanofluids. Hence, the results confirmed that the nanoparticles significantly improved the thermal stability of the insulating oil even in the presence of oxygen (air atmosphere); all nanofluids displayed a high degree of enhancement with maximum weight loss at high temperatures.

## 4. Conclusions

This research presented the influence of two as-synthesized semiconductive (CdS and Co_3_O_4_) nanoparticles on the AC dielectric strength and thermal stability of synthetic ester (Midel-7131) insulating oil for the first time. Nanoparticles of pure phase with high crystallinity, uniform morphology, and high specific surface area were successfully synthesized. When dispersed in Midel-7131, the results prove that SE/CdS (0.3 g/L) and SE/Co_3_O_4_ (0.05 g/L) nanofluids provided an enhancement of the AC dielectric strength by 31.9% and 31.3%, respectively. The formation of an electrical double layer owing to the high surface energy due to the large specific surface area and wide bandgap of the nanoparticles enabled the nanofluids to capture fast-moving charges and convert them to slow electrons, leading to a significant enhancement of the AC dielectric strength of synthetic ester oil. The addition of these nanoparticles to the synthetic ester oil induced an elongation of the hydrocarbon chains within the insulating oil, which led to enhanced thermal stability. The nanofluids exhibited reduced heat dissipation, signifying their improved thermal stability compared to the bare fluid. The statistical analysis proved that the experimental data followed both a Weibull distribution and a normal distribution. Furthermore, the minimum withstand voltage of the synthetic ester and nanofluid samples also provided satisfactory statistics. According to the statistical analysis, there was only a 1% breakdown probability of breakdown occurring for SE/CdS at 75.13 kV and SE/Co_3_O_4_ at 76.39 kV, verifying the safety and reliability of these nanofluids for power transformers.

## Figures and Tables

**Table 1:** Physiochemical properties of bare fluid (Midel-7131) [16].

Properties	Midel-7131
Density at 20 °C (kg/dm^3^)	0.97
Kinematic viscosity at 40 °C (cSt)	28–29
Pour point (°C)	−56
Flash point (°C)	260
Fire point (°C)	316
Total acid number (mg KOH/g)	<0.03 mg
Moisture content (ppm)	300
DC resistivity (GΩ·m)	>20
Dissipation factor at 90 °C	0.8%

**Table 2:** This mean AC breakdown voltage (AC BDV) and electric field strength (EFS) of synthetic ester (SE) and nanofluids.

Volume Fraction		Mean BDV (kV)	Increment (%)	EFS (kV/mm)
**Synthetic Ester (SE)**		**60.23**	**-**	**24.09**
**0.05 g/L**	**SE/CdS**	71.55	18.79	28.62
	**SE/Co_3_O_4_**	79.11	31.35	31.64
**0.2 g/L**	**SE/CdS**	75.45	25.26	30.18
	**SE/Co_3_O** **_4_**	74.42	23.56	29.77
**0.3 g/L**	**SE/CdS**	79.45	31.91	31.78
	**SE/Co_3_O_4_**	73.73	22.41	29.49
**0.4 g/L**	**SE/CdS**	76.33	26.73	30.53
	**SE/Co_3_O_4_**	72.95	21.11	29.18

**Table 3:** Conformity of average AC breakdown voltage to a Weibull distribution for synthetic ester and nanofluids.

Sample	*p*-Value	Conformity to Weibull Distribution
**Synthetic Ester Oil**	>0.250	**Accepted**
**SE-CdS-0.05 g/L**	<0.010	**Rejected**
**SE-CdS-0.2 g/L**	0.159	**Accepted**
**SE-CdS-0.3 g/L**	0.084	**Accepted**
**SE-CdS-0.4 g/L**	0.167	**Accepted**
**SE-Co_3_O_4_-0.05 g/L**	>0.250	**Accepted**
**SE-Co_3_O_4_-0.2 g/L**	0.049	**Rejected**
**SE-Co_3_O_4_-0.3 g/L**	>0.250	**Accepted**
**SE-Co_3_O_4_-0.4 g/L**	>0.250	**Accepted**

**Table 4:** Average AC breakdown voltage (kV) and increment (%) of different breakdown probabilities of various nanofluids and pure oil.

AC BDV Probability (%)		1		10		50	
Mass Fraction (%)		BDV	Inc	BDV	Inc	BDV	Inc
**SE Oil**		43.80	-	52.58	-	60.86	-
**0.05 g/L**	**SE/CdS**	57.99	32.40	65.31	24.21	71.83	18.02
	**SE/Co_3_O_4_**	76.39	74.40	77.97	48.28	79.25	30.21
**0.2 g/L**	**SE/CdS**	65.71	50.02	71.25	35.50	76.02	24.90
	**SE/Co_3_O_4_**	61.50	40.41	68.68	30.62	75.03	23.28
**0.3 g/L**	**SE/CdS**	75.13	71.52	77.57	47.52	79.58	30.75
	**SE/Co_3_O_4_**	63.83	45.73	69.41	32.00	74.23	21.96
**0.4 g/L**	**SE/CdS**	69.20	57.99	73.29	39.38	76.79	26.17
	**SE/Co_3_O_4_**	57.65	31.62	66.00	25.52	73.55	20.85

**Table 5:** Skewness and kurtosis of the AC breakdown voltage of synthetic ester and nanofluids.

Sample	Skewness	Kurtosis
**Synthetic Ester Oil**	−0.154	−0.568
**SE-CdS-0.05 g/L**	1.508	1.667
**SE-CdS-0.2 g/L**	−0.654	−0.900
**SE-CdS-0.3 g/L**	0.656	0.106
**SE-CdS-0.4 g/L**	−1.146	1.683
**SE-Co_3_O_4_-0.05 g/L**	−1.121	1.588
**SE-Co_3_O_4_-0.2 g/L**	−0.003	−1.719
**SE-Co_3_O_4_-0.3 g/L**	−0.381	−1.120
**SE-Co_3_O_4_-0.4 g/L**	0.025	−0.707

**Table 6:** Conformity to normal distribution of average AC breakdown voltage of synthetic ester and nanofluids.

Sample	*p*-Value	Conformity to Normal Distribution
**Synthetic Ester Oil**	0.625	Accepted
**SE-CdS-0.05 g/L**	<0.005	Rejected
**SE-CdS-0.2 g/L**	0.093	Accepted
**SE-CdS-0.3 g/L**	0.469	Accepted
**SE-CdS-0.4 g/L**	0.098	Accepted
**SE-Co_3_O_4_-0.05 g/L**	0.555	Accepted
**SE-Co_3_O_4_-0.2 g/L**	0.084	Accepted
**SE-Co_3_O_4_-0.3 g/L**	0.310	Accepted
**SE-Co_3_O_4_-0.4 g/L**	0.926	Accepted

**Table 7:** TGA–DSC–DTG results of synthetic ester oil and nanofluids in air and N_2_.

**In N_2_**						
**Samples**	**TGA**	**DSC**			**DTG**	
	**ΔY (%)**	**Onset Temperature (°C)**	**Endothermic Peak (°C)**	**Enthalpy (J/g)**	**Peak X (°C)**	**Peak Y (%/min)**
**SE**	98.879	334.2	349.94	469.505	349.08	−25.408
**SE/CdS**	100.108	334.43	353.94	408.147	353.69	−22.005
**SE/Co_3_O_4_**	98.547	335.26	358.30	405.8153	357.45	−23.572
**In Air**						
**Samples**	**TGA**	**DSC**			**DTG**	
	**ΔY (%)**	**Onset Temperature (°C)**	**Exothermic Peak (°C)**	**Enthalpy (J/g)**	**Peak X (°C)**	**Peak Y (%/min)**
**SE**	100.102	289.83/369.84	351.64/464.80	−648.4821	350.83	−19.737
**SE/CdS**	100.583	300.98	340.14	−732.0902	338.04	−21.672
**SE/Co_3_O_4_**	100.320	289.83	346.63	−775.6782	344.13	−20.161

## Data Availability

The data that support the findings of this research are available from the authors upon reasonable request.
